# Biocompatibility of Intraocular Lenses

**DOI:** 10.4274/tjo.10437

**Published:** 2017-08-15

**Authors:** Pelin Özyol, Erhan Özyol, Fatih Karel

**Affiliations:** 1 Muğla Sıtkı Koçman University Training and Research Hospital, Department of Ophthalmology, Muğla, Turkey; 2 Dünyagöz Hospital, Ophthalmology Clinic, Ankara, Turkey

**Keywords:** Uveal biocompatibility, capsular biocompatibility, Cataract surgery, hydrophilic acrylic intraocular lens, hydrophobic acrylic intraocular lens

## Abstract

The performance of an intraocular lens is determined by several factors such as the surgical technique, surgical complications, intraocular lens biomaterial and design, and host reaction to the lens. The factor indicating the biocompatibility of an intraocular lens is the behavior of inflammatory and lens epithelial cells. Hence, the biocompatibility of intraocular lens materials is assessed in terms of uveal biocompatibility, based on the inflammatory foreign-body reaction of the eye against the implant, and in terms of capsular biocompatibility, determined by the relationship of the intraocular lens with residual lens epithelial cells within the capsular bag. Insufficient biocompatibility of intraocular lens materials may result in different clinical entities such as anterior capsule opacification, posterior capsule opacification, and lens epithelial cell ongrowth. Intraocular lenses are increasingly implanted much earlier in life in cases such as refractive lens exchange or pediatric intraocular lens implantation after congenital cataract surgery, and these lenses are expected to exhibit maximum performance for many decades. The materials used in intraocular lens manufacture should, therefore, ensure long-term uveal and capsular biocompatibility. In this article, we review the currently available materials used in the manufacture of intraocular lenses, especially with regard to their uveal and capsular biocompatibility, and discuss efforts to improve the biocompatibility of intraocular lenses.

## INTRODUCTION

Biocompatibility is an important feature of intraocular lenses (IOL) which may influence their clinical performance in the short and long term. Biocompatibility may be broadly defined as the physical, chemical, and biological compatibility between a biomaterial and the body tissues, and the optimum compatibility of a biomaterial to the body’s mechanical behavior. Ideally, a fully biocompatible IOL is expected to exhibit the following features: elicits no foreign-body reaction, is accepted by the surrounding tissues, has good compatibility with the capsular sac, and provides satisfactory vision over the lifetime of the patient without any further intervention. Although the most important determinant of biocompatibility is the implanted IOL, biocompatibility is also affected by characteristics of the host and the surgical technique. However, the main features involved in the biocompatibility of IOLs themselves are the lens material properties, the optic edge design, lens surface properties, and haptic-optic combination.

Although the biocompatibility of IOL lenses should be evaluated as a whole, the biological impact of an implanted IOL is at the uveal and capsular levels. Therefore, IOL biocompatibility is classified as uveal and capsular biocompatibility.^1^ Uveal biocompatibility is determined by the inflammatory reaction to the IOL formed in the eye. Disruption of the blood-aqueous barrier during cataract surgery and IOL implantation results in a rapid inflow of protein and cells to the anterior chamber. The immediate consequence of this is protein deposition on the lens surface. This accumulation depends on the surface properties and chemical structure of the IOL material. Protein deposition on the IOL surface also facilitates the accumulation of other cells on the lens surface. Via activation of the compliment system, inflammatory cells are transformed into macrophage and giant cells, resulting in a foreign-body reaction against the IOL. This cellular response includes two different types of cells; the first are small, circular, fibroblast-like cells that peak in the first month, while the second are foreign-body giant cells that peak in the third month. The giant cells later degenerate and leave an acellular proteinous membrane on the IOL surface.^2^ Uveal biocompatibility is evaluated based on the aqueous flare resulting from these pathophysiological events and the cellular deposition on the IOL.

Poly(methyl methacrylate) (PMMA) is the first intraocularly implemented IOL material. PMMA has important advantages including very good tissue tolerance, low foreign-body inflammatory response, high uveal biocompatibility, relatively higher refractive index, and good optical properties.1 However, because of PMMA’s intolerance to high temperature and pressure and its rigidity, foldable IOLs are currently preferred.

The foldable IOLs used today pose no problems with regard to uveal biocompatibility when evaluated clinically. In all of the previous studies in the literature, the levels of aqueous flare and cellular deposition on the IOL surface were not clinically significant, and these studies focused only on comparing IOLs being used. It has been reported that cellular accumulation on the IOL was not clinically significant even in uveitic eyes, where uveal reaction may be more pronounced, in diabetic patients, and in eyes with pseudoexfoliation syndrome.^3,4,5,6,7^ Classifying the currently used foldable IOLs as hydrophobic and hydrophilic according to material properties revealed that hydrophilic IOLs have better uveal biocompatibility compared to hydrophobic ones.^8^ The better tissue compatibility of hydrophilic materials is due to the high water content. In a study comparing aqueous flare caused by a hydrophobic IOL and a heparin-coated hydrophobic IOL over a 3-month follow-up period, no significant difference was found except on the first postoperative day.^9^

Foldable silicone lenses have hydrophobic surfaces. Silicone lenses offer an advantage in terms of uveal biocompatibility because of the very low levels of cellular deposition on the IOL surface.^10^ In a study comparing hydrophilic, hydrophobic, and silicone IOLs, it was found that the amount of aqueous flare increased in all three types of IOL in the first month compared to preoperative levels; however, the amount of aqueous flare observed with the hydrophobic IOL was significantly higher than with the other types of IOLs. The authors reported that the amount of aqueous flare decreased after the first month and there was no significant difference among the IOLs during 18 months of follow-up.^6^

Early inflammatory cell deposition on the IOL is dominated by the small, circular cell type, which are an indicator of the blood-aqueous barrier disruption. The foreign-body giant cells which dominate later are an indicator of extended inflammation and are more responsible for uveal biocompatibility pathogenesis. Less inflammatory cell deposition occurs on hydrophilic IOLs compared to hydrophobic ones. It was found that accumulation of foreign-body giant cells predominates on hydrophobic IOLs, whereas accumulation of small circular cells is more prevalent on silicone IOLs. However, in long-term follow-up, it has been reported that there is no difference between IOLs with regard to cellular deposition.^4^

The pathogenesis of capsular biocompatibility involves proliferation and migration of lens epithelial cells. Lens epithelial cells form a single-cell lining beneath the anterior capsule and extend towards the equatorial lens curve. These cells exhibit mitotic activity, with maximum mitotic activity in the germinative zone encircling the pre-equatorial area of the anterior lens capsule. The newly formed cells proceed towards the equator, growing in volume and differentiating into a fibrillary structure. The epithelial cells located under the anterior capsule and those at the equator differ in function, growth pattern, and pathological processes. The lens epithelial cells under the anterior capsule do not proliferate, but rather exhibit fibrotic reaction. The cells in this area are the largest epithelial cells in the lens. The lens epithelial cells located on the equator tend to migrate along the posterior capsule in pathological cases and, instead of exhibiting fibrotic reaction, generally transform into large cellular structures called Elschnig pearls. Therefore, indicators considered in the clinical evaluation of IOL capsular biocompatibility are posterior capsule opacification resulting from the proliferation and migration of lens epithelial cells, anterior capsule opacification, or ongrowth of lens epithelial cells onto the anterior surface of the IOL.^11^

Posterior capsule opacification is the most common postoperative complication after successful cataract surgery and is the most important parameter of capsular biocompatibility. The development of posterior capsule opacification depends more on the optical edge design of the lens than on the IOL material. Studies have shown that a 360° sharp posterior optic edge significantly reduces posterior capsule opacification.^12,13,14,15^ The sharp posterior edge creates a barrier that prevents the advancement of lens epithelial cells along the posterior capsule. A meta-analysis evaluating 66 prospective, randomized, controlled studies compared IOLs of the same material with sharp and rounded edge designs and revealed that IOLs with a sharp-edge design lead to less posterior capsule opacification.^16^ In terms of IOL material characteristics, posterior capsule opacification occurs more frequently with hydrophilic compared to hydrophobic IOLs because a hydrophilic surface provides a foundation for lens epithelial cell proliferation and migration, whereas a hydrophobic surface adheres tightly to the posterior capsule due to its highly bioadhesive nature.^5,17,18,19,20,21^ Another important cause of posterior capsule opacification in hydrophilic IOLs is that their high water content does not allow as sharp a posterior edge as can be achieved in hydrophilic IOLs.^22^ Differentiation of lens epithelial cells from fibroblast-like cells causes opacification of the anterior capsule. This opacification is often clinically insignificant because it does not encroach on the optical axis. However, contraction of the capsulorhexis orifice as a result of fibrosis may cause IOL dislocation and associated refractive changes. With regard to material properties, the reported rate of anterior capsule opacification is lower for hydrophilic acrylic lenses compared to hydrophobic acrylic lenses.^23^ In terms of IOL optic edge design, it has been shown that contrary to posterior capsule opacification, a rounded or sharp posterior edge does not affect the degree of anterior capsule opacification.^24^ On the other hand, it was reported that the angled haptic-optic junction leaves a clearance between the posterior capsule and the IOL optic, which influences posterior capsule opacification.^23^ Although the removal of lens epithelial cells from under the anterior capsule during surgery could not be associated with the development of posterior capsular opacification, it has been shown to reduce anterior capsule opacification and contraction.^23,25^

The highly fibrotic reaction encountered in silicone IOLs is caused by lens epithelial cell proliferation being overstimulated by the silicone material. Dense fibrosis may result in posterior capsule opacification, anterior capsule opacification, and in some cases extreme fibrotic reaction which may cause IOL decentration.^11^ In addition, contact between silicone lenses and intravitreal gases and silicone oil causes the lens to lose transparency.

Ongrowth of lens epithelial cells onto the IOL occurs as a result of the proliferation of lens epithelial cells from the capsulorhexis edge towards the anterior surface of the IOL. It usually does not lead to opacification or vision loss. It is mostly seen in hydrophilic acrylic lenses.^26,27^

In parallel to the recent technological developments in cataract surgery, there have also been important advancements in IOL technology. IOL innovations include efforts to increase patients’ visual satisfaction, short- and long-term clinical performance, and IOL biocompatibility. Attempts to increase biocompatibility have often focused on modifying the surface or material properties of the IOLs.

Hybrid hydrophobic IOLs, developed by altering the properties of hydrophobic IOL material, have recently been introduced into clinical use. These hybrid hydrophobic IOLs are hydrophobic lenses that have a hydrophilic component. They have 4-5% water content and are stored in 0.9% saline solution. In a study comparing anterior capsule opacification scores in rabbit eyes with a hydrophobic IOL and a hybrid hydrophobic IOL over a 4-week follow-up period, a significantly lower anterior capsule opacification score was reported for the hybrid hydrophobic IOL.^28^ An important feature of this class of IOLs is that they exhibit practically no glistening formation, which is a problem encountered in hydrophobic IOLs.^29^ Glistenings are fluid-filled vacuoles within the IOL material. They are a consequence of the difference in refractive index that occurs when the crosslinks between hydrophobic IOL copolymers are filled with fluid. It usually does not affect visual acuity, but may have an impact on the quality of vision.^30^ It is believed that hybrid hydrophobic IOLs prevent glistening formation because they have tighter crosslinks and their hydrophilic structure provides water balance in the IOL material.

Other work aimed at improving IOL biocompatibility focused on changing the IOL surface properties. Considering the fact that hydrophobic IOLs have good capsular biocompatibility and hydrophilic surfaces have good uveal biocompatibility, several molecules are being used to add hydrophilic surface properties to hydrophobic lenses. IOL surface properties are often provided by surface treatment, surface coating, and adding molecules to the surface. Heparin is clinically used as a surface-coating molecule to increase biocompatibility. In previous years, it was used in the surface coating of PMMA lenses in order to improve biocompatibility in uveitic, diabetic, and pediatric patients with greater likelihood of postoperative inflammation. Studies have demonstrated that PMMA with heparin surface modification reduces early postoperative inflammation.^31,32,33^ Heparin surface coating gives the IOL surface a more hydrophilic character, thereby reducing inflammatory cell adhesion. Heparin coating of foldable hydrophobic IOL surfaces is also reported to effect changes in the clinical parameters of uveal biocompatibility.^9^

Various molecules have been used experimentally to imbue hydrophobic IOLs with hydrophilic surface properties. Polyethylene glycol is a molecule that increases uveal biocompatibility by reducing attractive forces between the lens surface and proteins.^34^ In another study, the posterior surface of a hydrophobic IOL was coated with N-vinyl pyrrolidone, a hydrophilic monomer, in order to obtain a hydrophilic posterior surface, and it was reported that the hydrophilic posterior surface increased uveal biocompatibility while the hydrophobic anterior surface increased capsular biocompatibility.^35^ Tissue growth factor beta-2 (TGF-β2) is an important factor in the stimulation of lens epithelial cells to form anterior capsule opacification. It was reported that a hydrophobic lens with anti-TGF-β2 surface modification both decreased lens epithelial cell ongrowth and increased lens surface hydrophilicity in experimental conditions.^36^

The biocompatibility features of IOL materials are summarized in Table 1 and the material properties of IOLs commonly used in Turkey are summarized in Table 2.

## CONCLUSION

Biocompatibility is an important property of an implanted IOL which reflects its long- and short-term clinical performance. With regard to IOL material properties, a sharp-edged anterior optic design and a hydrophobic surface are important for capsular biocompatibility, while a hydrophilic anterior surface is important for uveal biocompatibility. However, as the uveal biocompatibility of current foldable IOLs is not an important clinical problem even in the majority of eyes with higher risk of inflammation, it seems more clinically meaningful to prioritize capsular biocompatibility. While the material, surface properties, and optic design of IOLs are the main factors determining biocompatibility, other host and surgical factors should also be considered. Therefore, instead of choosing a single IOL with ideal biocompatibility for all patients, biocompatibility should be evaluated separately for each patient, also taking into account the nature of the planned surgery.

## Figures and Tables

**Table 1:**
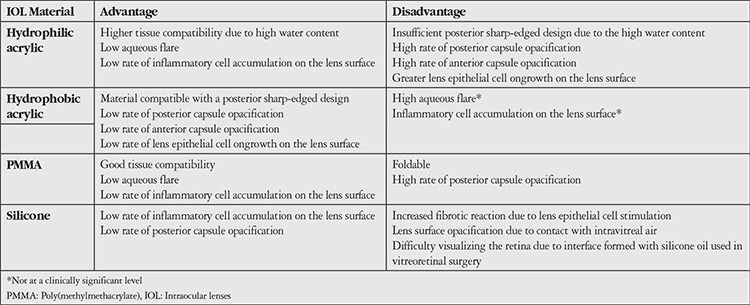
Comparison of the biocompatibility of intraocular lens materials

**Table 2:**
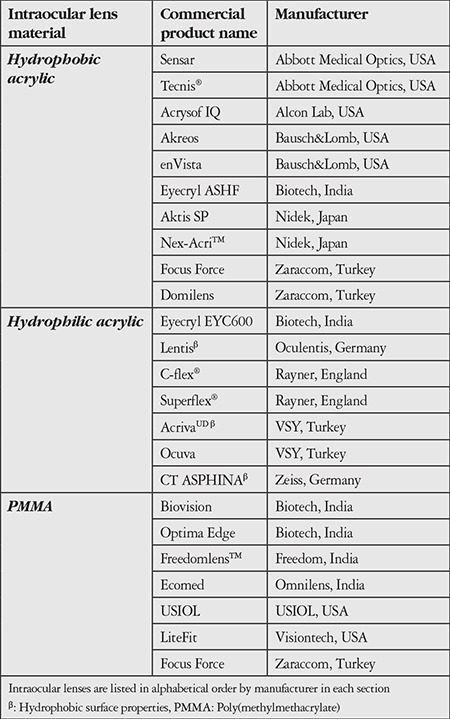
Intraocular lenses commonly used in Turkey and their commercial names*
